# A League of Mercy in the Colonies

**Published:** 1904-07-23

**Authors:** 


### A LEAGUE OF MERCY IN THE COLONIES.

THE serviceable "Hospitals' Volunteer Help League" of Melbourne was started in 1903 as an entirely new and original experiment without communication with the League of Mercy of London, of which nothing was known here except what could be gathered from brief mentions in newspapers. One difference between the Leagues is that whereas the members of the League of Mercy undertake to collect 20s. per annum, the voluntary collectors of the Melbourne League undertake to call every month for donations, however small. The Hospitals' Volunteer Help League commenced work in September 1903, and now has in hand £250, the result of very small monthly donations, averaging, it is said, about 8d. each member. The League began with two branches, and ultimately hopes to have as many as 36 branches in the Metropolitan area. In each district where a branch is formed a free public lecture on Hospitals and Hospital Work is given, illustrated with lantern slides. The originator of the idea here, Mr. F. W. Maudsley, was elected first president. He hopes that, when the first annual report is presented and the public realises what a valuable auxiliary to maintenance of the hospitals this mode of collecting at infinitesimal cost could be made, the support will become much more general. Mrs. T. R. Andrews, wife of the Secretary of the Melbourne Hospital, acts as honorary lecturer to the League.

